# Automatic Identification and Visualization of Reaction Mechanisms Contained within Direct Dynamics Simulations

**DOI:** 10.1021/acsomega.5c04493

**Published:** 2025-09-24

**Authors:** Trent Kobulnicky, Emmanuel Boafo, George L. Barnes

**Affiliations:** Department of Chemistry, 6049 Illinois State University, Campus Box 4160, Normal, Illinois 61790-4160, United States

## Abstract

Direct dynamics simulations are employed in many areas of chemistry and biochemistry. When paired with an appropriate underlying *ab initio*, semiempirical, or DFT-based potential energy surface and proper sampling of initial conditions, direct dynamics simulations provide an atomic-level view of the reaction dynamics within the system of interest, yielding considerable fundamental insights. Moreover, when a sufficient number of simulations are conducted, they provide a wealth of information regarding the overall trends in reactivity. However, they also generate large data sets that often require significant manual interpretation through inspection or developing case-specific analysis techniques. Here, we present an analysis method using a multitiered graph theory approach, which automatically highlights the most important mechanistic steps present within an ensemble of direct dynamics simulations. The effectiveness of this approach is demonstrated by examining results from three direct dynamics data sets previously reported for systems relevant to the tandem mass spectrometry community.

## Graphical abstract

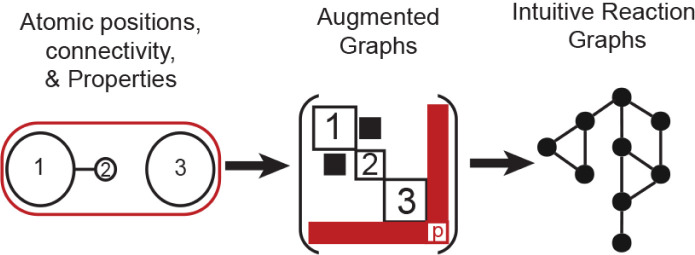

## 1 Introduction

Direct dynamics simulations have a long history^1–3^ and there is no doubt of the importance and success of the direct dynamics simulation methodology as highlighted in recent review articles.^4,5^ Direct dynamics simulations provide atomic-level insight into many systems’ physical and chemical processes. In particular, direct dynamics simulations have a long and successful history of providing the community with valuable insight regarding energy transfer and chemical reactivity.^6–8^ Direct dynamics have revealed unexpected mechanisms, such as round-about SN2 reactions,^9^ “shattering” or other fast fragmentation events^10,11^ and “roaming” mechanisms.^12–15^ Several reviews have been written that highlight the utility of this approach within the mass spectrometry community, with a particular focus on elucidating the energy transfer and reaction dynamics that take place.^16–19^ Multiple examples of insight gained from surface-induced dissociation (SID)^10,11,14,20–26^ and collision-induced dissociation (CID)^12,13,15,27–38^ simulations are present in the literature.

By their very nature, direct dynamics simulations provide a significant amount of information. Assuming a gas-phase system with *N* atoms, a typical simulation will, at the very least, provide 3*N* positions and 3*N* momenta per time step recorded. Investigators can “observe” this data using visualization tools such as VMD^39^ or PyMol.^40^ Once investigators gain insight into the types of processes and chemical reactivity that occur within their system of interest, these 6*N* data points per time step recorded can be used in creative ways to create derived data (such as internal coordinates) that, along with system-specific criteria, help provide insight into how chemical and dynamical processes take place. This approach, while effective, often relies heavily on human intuition, past experience, and direct inspection, all of which can introduce bias into the analysis. In addition, these approaches require a significant time investment related to direct inspection and the development of one-off analysis approaches for each relevant reaction pathway. Graph theory can be applied in many different ways to aid in the analysis of molecular dynamics simulations and enable a systematic workflow.

Gaigeot and coworkers^41,42^ have used graph theory to examine molecular dynamics simulations and extract information regarding the conformations explored by the system. In their approach, they encode not only information regarding covalent bonds but also hydrogen bonding and electrostatic interactions with ions. By including this additional information directly into the graph, they can study transitions between various conformations and isomers and examine how long each chemical species was visited during the trajectory, which provides valuable insight. Ozkanlar and Clark^43,44^ have also developed an approach to encoding noncovalent interactions into a graph-based framework. Their tools, MoleculaRnetworks and ChemNetworks, allow for the analysis of topological networks, such as solvent organization, from molecular dynamics simulations.

Other works have also explored the use of molecular dynamics simulations to quantify reaction networks. For example, Tsutsumi et al. outlined an on-the-fly trajectory mapping method^45^ and the reaction space projector (ReSPer) method.^46^ In combination, these techniques enable direct dynamics simulations to be mapped onto a series of intrinsic reaction coordinate (IRC) pathways along with an analysis of how a direct dynamics simulation traverses reference structures throughout time. This provides insight into how the trajectory traverses the reaction network in terms of these important reference structures. Maeda et al. have also outlined a means of exploring a reaction network automatically through the Artificial Force Induced Reaction (AFIR) method.^47^ The AFIR method allows for the automatic determination of reaction steps through the application of forces to groups of atoms to induce a chemical transformation. AFIR has been successfully applied to map out catalytic cycles without the use of any assumptions.^48^ The approaches of Tsutsumi and Maeda are complementary in that the AFIR approach could be used to obtain the IRCs that would be used for trajectory mapping and ReSPer. While the above approaches are focused on examining chemical reactivity, graph theory can also be used to analyze molecular dynamics simulations to analyze overall structure and interconnectivity. Concurrently, the work of Martinez–Nunez and coworkers has illustrated the great utility of performing high-energy, short-time direct dynamics simulations to explore the chemical space available to a system, which has led to the development of AutoMeKin.^49–51^ AutoMeKin provides an automatic way of mapping out reaction pathways and provides a graph of the chemical reactivity. However, graphs of chemical reactivity quickly become quite large, making them difficult to visualize efficiently. While follow-up kinetic Monte Carlo calculations provide long-time “equilibrium” results, they would provide a purely statistical view of the important reaction pathways.

The work of Perez-Mellor and Spezia provides a very nice overview of some of the most natural and direct applications of graph theory to the data supplied from direct dynamics.^52,53^ Here, we outline an extension to the molecular graphs initially described by Perez-Mellor and Spezia to enhance the analysis of nonequilibrium structures. Specifically, we propose including relevant chemical properties directly within the graph representation. While this approach shares some similarities with that of Gaigeot’s topological graphs, which uses both directed and undirected edges to denote the type of chemical interaction, it is unique in that nodes are introduced into the graph to represent collective chemical properties that are held by a subset of atoms within the system. Performing an analysis of the time evolution of the molecular graphs within this extended framework allows for an ensemble reaction graph (ERG) to be formed that automatically includes information regarding each pathway’s relative importance. Our ERG is similar to what Gaigeot terms a transition graph, although they each display different annotations and information. Graphs of reaction networks are often quite complex and quickly become challenging to visualize. To address this issue, the information contained within the ERG is used to identify and visualize the most important pathways that lead to a given product of interest and we illustrate the utility of this method by reexamining three previously published systems that were analyzed initially using more traditional approaches.

## 2 Methods

The application of graph theory to chemistry problems has been established.^54^ Since both chemical structures and reaction networks can be represented as graphs, graph theory can be applied to chemistry in many different ways. The analysis approach presented here uses both chemical structure graphs and reaction network graphs based on direct dynamics simulations. Below, we outline our method for forming and extracting information from each type of graph. Moreover, we propose an extension of the standard chemical structure graph that encodes information about the associated properties in a novel way. Lastly, we briefly outline the methods used to obtain the previously described data sets that we use here to illustrate this analysis technique’s effectiveness.

### 2.1 Augmented Molecular Structure Graphs

Perez-Mellor and Spezia recently provided a well-presented formal approach to applying graph theory to analyze direct dynamics simulations.^52^ We were inspired by that work and took it as our starting point. Here, we provide a brief outline of the most relevant portions of their analysis framework and encourage readers to see their original work for greater details. Given a set of *N* direct dynamics trajectories, the resulting data can be stored in a series of generic containers such that **X**[*i*, *j*] denotes the container that contains information for the *i* ^*th*^ trajectory (*i* ∈ [1,*N*]) at the *j* ^*th*^ point in time. We note that these storage containers are sized appropriately for their data type. For example, using this nomenclature, **XYZ**[*i*, *j*] would represent a set of Cartesian coordinates (i.e., a 2D array) from the *i* ^*th*^ trajectory at the *j* ^*th*^ point in time.

With the raw data appropriately stored, the derived data can be calculated. As outlined by Perez-Mellor et al., a molecular geometry, such as **XYZ**[*i*, *j*], can be converted into an undirected, κ-colored graph where each atom corresponds to a vertex and each bond corresponds to an edge connecting the corresponding vertices. Each vertex is assigned a unique color according to its atom type, e.g., C, H, N, etc. Such a graph can be constructed using simple distance-based cutoffs, as was done by Perez-Mellor et al., or it could be formed using a bond order cutoff, either by calculating bond orders from **XYZ**[*i*, *j*] or from a bond order matrix, **BO**[*i*, *j*], that was saved during the trajectory. Practically speaking, constructing an adjacency matrix is one of the most straightforward ways of forming this graph. In graph theory, a pair of vertices is considered adjacent if an edge connects them. Hence, an adjacency matrix is a (0,1)-matrix consisting of zeros along the diagonal and 1’s on off-diagonal elements that correspond to adjacent vertices. Using the established nomenclature, this matrix could be stored as **ADJ**[*i*, *j*]. With both the adjacency matrix and the colors of each vertex, the canonical label of the κ-colored graph is obtained through a graph isomorphism algorithm, which has been implemented in the NAUTY package, in particular the *amtog* and *labelg* utilities.^55^

These canonical labels uniquely identify the underlying connectivity of the atoms within a system of interest while also automatically including permutation invariance among chemically indistinguishable atoms. This method is general and can be performed at any point in time. However, since it is based on forming an adjacency matrix from distance (or bond order)-based cutoff values, it has the potential to “mislabel” species when they are far from their final exit pathway, i.e., far from an equilibrium configuration. Mislabeling could occur during dissociation or rearrangement events, especially when there is a change in a chemical property, such as during charge transfer. Since the graph is formed from cutoff values related to either distance or bond order, it is easy to believe that if, during a trajectory, such a graph contains disconnected components (i.e., the system dissociates), it would be possible for the properties within the system (i.e., charges) to either advance or lag behind that dissociation event and potentially do so in different ways for different trajectories. We have observed mislabeling related to such processes in our raw data. From a practical point of view, it could be argued that determining consistently performing cutoff values on the basis solely of distance or bond order as a proxy for the behavior of the property, while potentially possible, is not the simplest solution for obtaining the information needed to analyze the chemistry taking place.

In short, the potential mislabeling does not have to do with the available information; instead, the issue arises since the graph analysis employed only uses coordinates (bond distances) or bond orders to assign the edges between vertices. However, there may also be other associated information available for the relevant properties. While this information could be included as a property of an individual node, the relevant properties are not used to determine the edges present between the nodes. One way to overcome this challenge is to introduce collective properties directly into the graph. Hence, we introduce the term “atomic vertices” to refer to those related to the coordinates of the atoms and “collective property vertices” to refer to abstract collective properties of interest in the system. We note that from a graph theory point of view, there is no difference between these nodes and that the terms we introduce are to allow chemists to quickly grasp the significance of each node. The atomic vertices can still have properties associated with them, such as partial charge or Cartesian coordinates, and can be connected with edges according to established approaches. The collective property vertices serve to augment this information without changing it. The presence of collective property vertices allows for the chemical bonding to be preserved within the edges connecting the atomic vertices, while the edges between the atomic vertices and the collective property vertices provide new information. However, it is likely that these collective properties would be determined by using information contained within the atomic vertices. The collective property vertices are assigned their own distinct color, and the addition of edges between the collective property nodes and atomic nodes provides the graph with information about which connected or disconnected component(s) of the graph holds that property.

The above description is abstract, and as such, in this section, we provide an example for a system with an overall 1+ charge state. The graph would be formed by treating the atomic vertices and the edges between them using Perez-Mellor et al. approach. An additional collective property vertex, the 1+ charge state collective property vertex, was added with a unique color. In order to accomplish this, we assume that the spatial charges of each atom are available along with the connectivity of each atom. Hence, the total charge of each fragment, i.e., each disconnected component, can be determined using a simple sum. If the system has a total charge of 1+, then the collective property could be assigned to the disconnected component with a total charge within ± 0.1 of unity. If no single disconnected component had a sum of partial charges close to unity, then sums of total charges among previously connected disconnected components could be examined to identify the correct assignment of this collective property. Such an event would occur in the midst of a chemical change, and hence, the history of the trajectory would allow for the identification of which disconnected components were previously connected. Edges would then be added between the collective property vertex and the appropriate atomic vertices, i.e., those vertices included in the disconnected components with a sum of partial charges within ± 0.1 of unity. Within this framework, two timesteps from a simulation could have an identical atomic subgraph, but the 1+ property could be associated with different disconnected components of the atomic subgraph as needed. Ultimately, this results in the formation of what we term the augmented adjacency matrix, **AADJ**[*i*, *j*], for each trajectory and time step. Again, this is one example of a simple case of a system with a 1+ charge. If the system had a 2+ charge, collective property vertices of 1+ and 2+ could be included with similar sums of partial charges to determine appropriate additional edges to include in the graph. Two collective property vertices would be needed to allow for the possibility of fragmentation products with either a 1+ or 2+ charge state.

This approach is illustrated in Figure 1. We note that both the standard and augmented canonical labels are displayed for illustrative purposes only; in the actual implementation of the approach, only the augmented canonical labels would be calculated. Column 1 schematically shows a system with three atomic components. Components 1 and 2 are connected in the first frame, while component 3 is disconnected. The first two components hold a collective property in the first frame, indicated by the red box surrounding them. Moving down this column, we see component 2 disconnecting from component 1 and connecting to component 3. The second and third cells of column 1 illustrate that component 2 may disconnect from component 1 before the collective property starts to be shared with component 3 and that there is an intermediate state in which atoms of all three components share the collective property. The dotted line between components 1 and 2 represents the potential of using different cutoff values and/or modifying the atomic adjacency matrix based on physical arguments. For example, if component 2 is a single atom, it may not make sense for it to be unbound; instead, it should always be connected to other atoms. The second column of Figure 1 schematically represents the augmented adjacency matrix and provides associated canonical labels for the atomic adjacency matrix, the augmented adjacency matrix, and relevant versions of these matrices with edges corresponding to the dotted lines in column 1, if applicable. Only the augmented version of these canonical labels distinguishes all of these examples. While Figure 1 is greatly simplified, it is directly related to charge-transfer processes involving the movement of a H^+^ atom, commonly occurring within tandem mass spectrometry.

**Figure 1:**
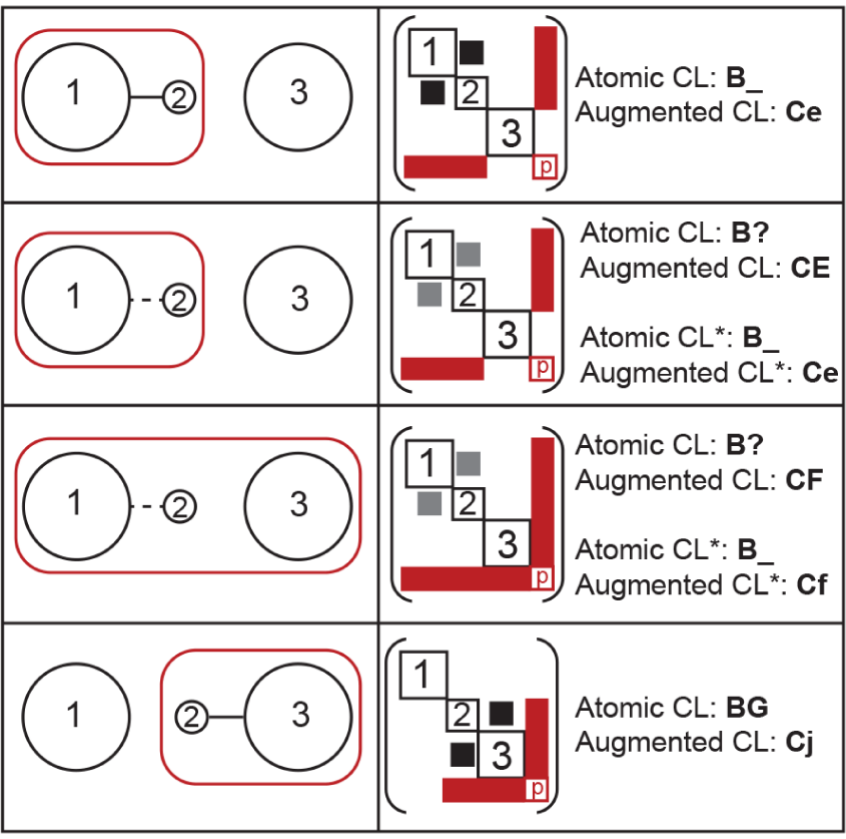
Schematic representations of a system with three “atomic” components and one collective property. Column one illustrates various system states, while column two provides schematic representations of the augmented adjacency matrix and several canonical labels (CLs). The atomic CL represents labels that consider just the atomic components and edges, while the augmented CL considers the full matrix shown. Any label with an asterisk represents a modified atomic adjacency matrix with edges shown as dotted lines in column 1 and illustrated as gray boxes in column 2. The use of property nodes allowed these states to be distinguished.

### 2.2 Construction of Ensemble Reaction Graphs

In the previous section, we described a graph-theory-based approach to analyze individual frames from direct dynamics simulations that incorporated chemical properties in the graph. Here, we describe how graph theory can be applied to determine the most important reaction pathways in an ensemble of trajectories. In particular, the analysis from the previous section provides the **AADJ**[*i*, *j*] from which the augmented canonical labels, **ACL**[*i*, *j*], are obtained. For each trajectory, *i*, a time series analysis of these data allows for the important times and the corresponding state of the system to be recorded. This can be accomplished by performing isomorphism tests between time steps or directly analyzing ACLs. Performing isomorphism tests between frames will add computational expense. While isomorphism tests are believed to reside in the NP-intermediate class, in real-world practical applications, there are several efficient algorithms available^55^ Any fast oscillations between states can be removed, such that artifacts from high-frequency stretching motions are not considered in the final analysis. The resulting series of ACLs, termed **S**[*i*], provides a record of the important mechanistic *steps* that took place for the *i* ^*th*^ trajectory.

Unlike in the previous section, where a graph was formed from atoms and properties, here we describe a graph in which each ACL visited during a trajectory is considered as a vertex with edges connecting the vertices obtained from the steps within **S**[*i*]. Performing this analysis for every trajectory results in what we term an ensemble reaction graph (ERG). At a basic level, the ERG consists of all unique ACLs (graph vertices) and ACLs that are interconnected (edges). The direct dynamics simulations provide sufficient information to allow for each interconversion to be denoted as a reversible or irreversible step (i.e., the edges are directed) along with the relative importance of each interconversion. However, it is important to note that in this context, the term reversible and irreversible only refers to whether the reverse event was contained within the ensemble of trajectories. If an edge is identified as irreversible, it does not necessarily mean that, chemically speaking, the process is truly irreversible, just that it does not reverse within the data set provided. The ERG is similar to the reaction network graphs generated by AutoMeKin. However, that framework provides energetics between states, while the frequency of moving between states is available in the ERG. We note that an ERG may contain some nonequilibrium states that happen to be transition states; however, finding all transition states is not an explicit goal of this approach.

As an aside, while canonical labels encode an entire (augmented) atomic graph and provide a wealth of information, they are difficult for humans to interpret quickly. It is therefore recommended to create an isomorphic mapping between a set of human-readable labels that also provide insight into the system and the complete set of unique ACLs explored by the ensemble of trajectories. As an example, for CID systems, labeling based on the *m*/*z* of the system along with an isomer number is both human-readable and provides immediate insight into the state of the system. We implement this particular isomorphic mapping in the examples shown below.

Reaction network graphs can become overwhelming and difficult to interpret directly. In the present approach, even a single occurrence of a new ACL in one trajectory introduces a new edge and vertex to the ERG. Some filtering must occur to obtain insight into the most relevant pathways observed within the direct dynamics simulations. Hence, the remainder of this section is devoted to describing a method of defining a *view* of the ERG that provides insight into the most pertinent dynamic steps resulting in the products of interest. Qualitatively speaking, this approach begins with identifying the products of interest, a set that we term **P**. This set may contain one or more members, depending on the selection criteria. In the absence of large stretches, every direct dynamics simulation’s initial state within an ensemble is known and identical between trajectories. We will term the initial ACL as *I*. Hence, the task is to find the most important pathways between the initial state *I* and all states within **P**. Toward this end, we employ a breadth-first search algorithm starting with the products of interest. Namely, each member of **P** is analyzed to determine the edges that end in that state. The most relevant of those edges are identified and saved. The starting points of these newly identified edges are added to the set of most relevant ACLs and, in turn, analyzed. This recursive process continues until no more edges or ACLs are added. Below, we outline the technical details of this approach.

Throughout the following discussion, directed edges will be denoted as *E*_*i→j*_ where *i* and *j* are ACLs. It is helpful to introduce the normalized incoming flux function, which provides a normalized contribution to the incoming transient population of ACL *j* from edge *E*_*i→j*_. This is a transient population since it considers the net incoming flux but does not consider the final population in each ACL. This function is defined as

1 $$F\left(E_{i\to j}\right)=\left\{ \begin{array}{ll} \frac{1}{N_{j}}\left[C\left(E_{i\to j}\right)-C\left(E_{j\to i}\right)\right], & \mathrm{if}C\left(E_{i\to j}\right)>C\left(E_{j\to i}\right)\\ 0, & \mathrm{otherwise} \end{array}\right.$$

where *C*(*E*) is the count of occurrences within the ensemble for a given directed edge, and *N*_*j*_ is a normalization constant such that ∑_*i*_ *F*(*E*_*i→j*_) = 1. The normalized incoming flux function can be used to directly rank the relative importance among all edges that lead to a given vertex, i.e., the chemical state of the system, within the ERG.

System-specific criteria allow for the determination of the final products that one wishes to explore. These final *products* form set **P**. To initialize the breadth-first search, the *relevant* set of ACLs, denoted as **R**, is initially defined as identical to **P**. When analyzing the *k* ^th^ member, *R*_*k*_, we start by identifying the set of all edges connected to *R*_*k*_, i.e., *E_i_* →*R_k_*, which we denote as **E** ^**t**^, a temporary set that contains potential additions to this view of the ERG. To filter the ACLs and edges displayed in the current view of the ERG, the most relevant subset of **E** ^**t**^ is determined based on the following steps:

1) The normalized incoming flux function is calculated, and **E** ^**t**^ is sorted from largest to smallest contribution to $N_{R_{k}}$ .

2) Members of **E** ^**t**^ below $F_{c}$ , an adjustable parameter denoting the minimum contribution to $N_{R_{k}}$ that should be displayed, are removed from consideration.

3) The remaining members of **E** ^**t**^ are retained within a new subset, **E** ^**t′**^, such that **E** ^**t′**^ simultaneously contains both the members of **E** ^**t′**^ that contribute the most to $N_{R_{k}}$ and has the minimum number of members necessary to explain at least $P_{c}$ of the normalized incoming flux. Hence, this new set will satisfy:

2 $$P_{c}\leq \overset{E^{t^{\prime }}}{\underset{i}{\sum }}F\left(E_{i\to R_{k}}\right)$$

Note that if two edges have the same value for the normalized incoming flux function, both are included in **E** ^**t′**^.

For some *R*_*k*_, the conditional sum in eq 2 cannot be met if many small contributions are removed in Step 2. This typically occurs for ACLs with a small number of occurrences within the ensemble of trajectories. With **E** ^**t′**^ identified, all *E* ∈ **E** ^**t′**^ are added to the set of relevant *edges*, **E**. In addition, every ACL $i\in {E^{t}}_{i\to R_{k}}^{\prime }$ are added to the set of relevant vertices, **R**. This procedure continues iteratively until all members of **R** have been analyzed. We note that the starting ACL, *I*, is not analyzed, but edges that start at *I* will be included.

In summary, this approach results in a view of the ERG that provides insight into which dynamic steps are most important for obtaining the products of interest, **P**. The adjustable parameters $P_{c}$ and $F_{c}$ are utilized to generate the set of the most relevant ACLs **R** intermediate between the starting configuration and **P**. The set **E** contains the relevant edges that are needed to form a connected graph that links *I* → **R** → **P**. Multiple unique pathways are obtained simultaneously, provided that the edges involved are considered important based on the parameters $P_{c}$ and $F_{c}$ . The parameter $P_{c}$ specifies the target netflux to be explained, while $F_{c}$ specifies the minimum netflux required to be considered important. The most commonly occurring pathways are immediately apparent with this approach.

## 3 Results and Discussion

The approach described in the methods section ultimately results in an ensemble reaction graph (ERG) and one or more views of that ERG that provide insight into products of interest. This method is applicable to many systems. The Barnes Research group, a primarily undergraduate research group, studies the reaction dynamics occurring in high-energy collision systems relevant to tandem mass spectrometry. Here, we illustrate the utility of this approach by applying it to three previously calculated ensembles of trajectories,^33,35,37^ aimed at modeling argon CID. The particular CID systems that were studied and revisited here are protonated *O*-sulfonated serine,^33^ lysine-H+^35^ and acetyl-lysine-H^+^.^37^ In all cases, trajectories were calculated using standard methods.^17^ Namely, for those systems that included an explicit collision with Ar, once the minimum energy structure was obtained, the initial internal energy was sampled from a 300 K Boltzmann distribution for both vibrations and rotations.^56^ A random orientation and impact parameter were selected, and a specified relative collision energy was chosen. Initial internal energy was sampled from a microcanonical ensemble for vibrational energy for those systems with an implicit collision.^57^ Simulations were integrated using Hamilton’s equations of motion using a 6^*th*^-order symplectic integration scheme^58^ with a 1 fs time step. Output was recorded every 50 fs, and a sufficiently long simulation time was used to sample the system’s reactivity. Additional system-specific information regarding the collision systems and the data sets will be outlined in their associated subsections below.

### 3.1 Method Implementation

To implement this approach, an in-house Python code is employed to obtain **S**[*i*] through the use of the iGraph package.^59,60^ The important times are located by examining the isomorphism between the graphs represented by **AADJ**[*i*, *j*] for all recorded time steps. Our examples are CID systems with species holding a 1+ charge. Hence, the collective property included in the graph is the 1+ formal charge state. The standard **ADJ**[*i*, *j*]­s are formed using the stored **BO**[*i*, *j*], while the collective property vertex is added using the partial charges, **Q**[*i*,*j*]. These partial charges are available from the semiempirical calculations used in the direct dynamics simulation at each time step. Once the **S**[*i*] are identified through isomorphism tests between frames, the corresponding edges (*S*_0_[*i*] → *S*_1_[*i*], *S*_1_[*i*] → *S*_2_[*i*], etc.) and unique ACL’s for all trajectories in the data set are stored in an SQLite3 database. Isomorphism tests are performed using the BLISS algorithm^61,62^ implemented in the iGraph package. This database could include multiple ensembles simultaneously, each with different selections for the initial conditions.

Analysis of this database produces **P**, **R**, and **E**. The set **P** is determined from the most populous final *m*/*z* peaks and the isomers with the largest population within those peaks. If the view of the ERG became too complex to examine all of **P** simultaneously, a subset of **P** is selected. A view of an ERG could be generated for a single ensemble or multiple ensembles simultaneously, with results annotated to provide that information.

Graphviz and the dot language^63^ are employed to visualize each view of the ERG with each directed edge labeled by its normalized incoming flux function for the ending vertex along with the net total counts for that edge, i.e., *C*(*E*_*i→j*_) - *C*(*E*_*j→i*_). Some ACLs are connected reversibly, i.e., both *C*(*E*_*i→j*_) and *C*(*E*_*j→i*_) are nonzero. To decrease the number of edges displayed and increase the readability, a single edge is drawn in black when two ACLs can interconvert. In contrast, irreversible transformations are drawn with a red edge. Each edge is labeled with information corresponding to the more frequently occurring direction, which is indicated by the edge’s arrowhead.

Rather than just labeling nodes in the ERG with isomorphic mapping of the ACLs, we use additional information from direct dynamics simulations. Namely, example coordinates for each ACL are obtained and processed with RDKit^64^ using Jensen’s implementation of Kim and Kim’s algorithm.^65^ Once the ACLs are represented as RDKit molecules, they are output as graphical Lewis structures. We note that the Lewis structures generated by RDKit for some nonequilibrium structures are not perfect. This is unsurprising given that RDKit’s routines are designed to work on minimum-energy structures, and converting Cartesian coordinates to RDKit molecules is already challenging. However, despite some imperfect Lewis structures, this approach automatically analyzes a large data set and highlights important reaction steps that researchers can refine further, as needed. For all of the systems considered below, we choose $P_{c}=0.85$ such that each node in a given view of an ERG accounts for close to 85% of the net incoming transient population. For the systems considered here, we choose $F_{c}$ to be a small value such that any event that takes place more than two times could be included in the view of the ERG. In practice, for the systems considered here, it is seen that in most cases we reach $P_{c}\approx 0.85$ well before the cutoff of $F_{c}$ becomes relevant, but this may not be true for all systems considered. For the ERGs shown below, our aim is to explain the product ions seen at the end of the simulations. Toward that end, we examine *m*/*z* peaks that were considered significant in the previously reported work and focus on product ions within those peaks that are frequently observed, e.g., account for a fraction of ∼0.05 or more. Again, we emphasize that the views of the ERGs presented below are obtained automatically without additional human input or adjustment.

### 3.2 *O*-Sulfonated Serine + Ar CID

Sulfonation is a post-translational modification that occurs for serine. The CID of N-terminus protonated *O*-sulfonated serine (s-Ser-H^+^ - [C_3_H_8_NO_6_S]^+^ - *m*/*z* 186) was studied experimentally^66^ and computationally by Lucas et al.^33^ Lucas et al. modeled its CID using direct dynamics simulations employing the PM6 semiempirical method to treat the potential energy of s-Ser-H^+^ and literature values for the force field parameters to model the explicit collision with Ar at a range of relative translational energies. In agreement with experiment, the primary decomposition pathways were found to be the loss of SO_3_ to form *m*/*z* 106 ([C_3_H_8_NO_3_]^+^) and the loss of H_2_SO_4_ (or H_2_O + SO_3_) to form *m*/*z* 88 ([C_3_H_6_NO_2_]^+^). While in the original work a range of collision energies between 2 and 11 eV was considered, here, we form an ERG for the collision energies of 8 and 9.5 eV. Based on our prior work, we anticipated that most, but not all, of the products for *m*/*z* 88 will be found, while all of *m*/*z* 106 will be present. For illustrative purposes, Figure 2 shows the graph for the ensemble of trajectories, with every edge labeled by the number of times it occurs in the ensemble. As is evident, such a graph does not readily provide insight into the dynamics within the system and is of little use in analysis.

**Figure 2:**
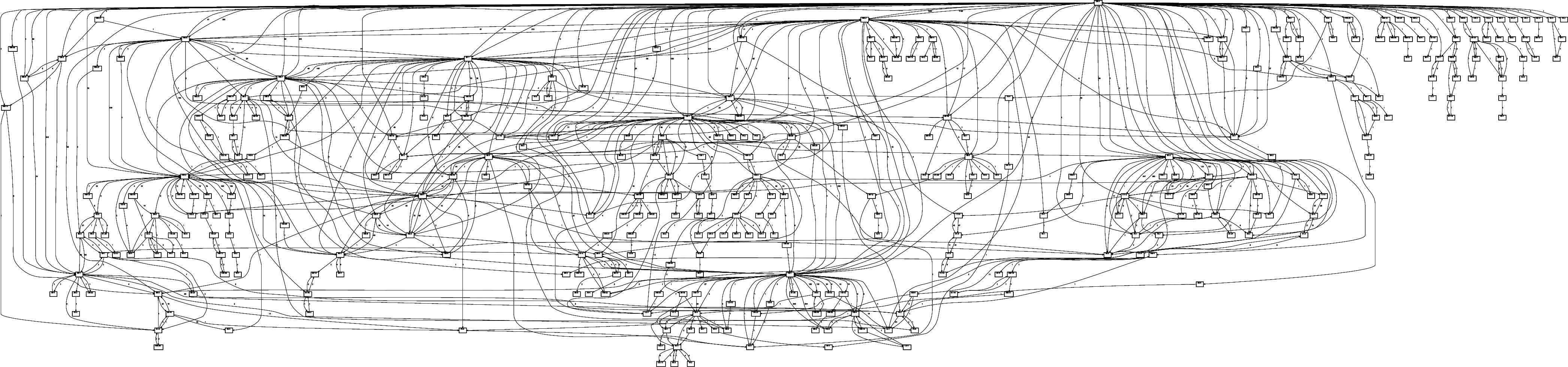
A graph showing all the nodes and edges present for the ensemble of trajectories in the s-Ser-H^+^ system for collision energies of 8 and 9.5 eV. Interpreting this graph is challenging due to the large number of transitions within the data set. This graph visually represents the “raw data” used to construct the views of the ERG shown below.

We start our analysis with the *m*/*z* 106 peak and obtain the view of the ERG shown in Figure 3, without any additional human intervention other than setting the desired width of the plot. It is advisable to analyze views of ERGs from the bottom up. A comparison of each incoming edge immediately highlights the relative importance and allows for a straightforward means to trace the most important pathway back to the starting ACL. From the figure, there is a single structure that accounts for 97.7% and 95.2% of the *m*/*z* 106 population at a collision energy of 8 and 9.5 eV, respectively. This view of the ERG also makes it clear that several pathways result in this final structure; 73% of the incoming flux is through a mechanism in which SO_3_ and Ser-H^+^ are formed directly. This can also occur by forming an intermediate of ${\mathrm{HSO}}_{3}^{+}$ through a direct loss of this intermediate or through a different protonation state of the starting material, labeled 186–7. These results are in agreement with Lucas et al.^33^ It is observed that when the product is directly formed from the starting configuration, it is an irreversible process, whereas the formation of the intermediates (186–7 and 81–1) is a reversible steps.

**Figure 3:**
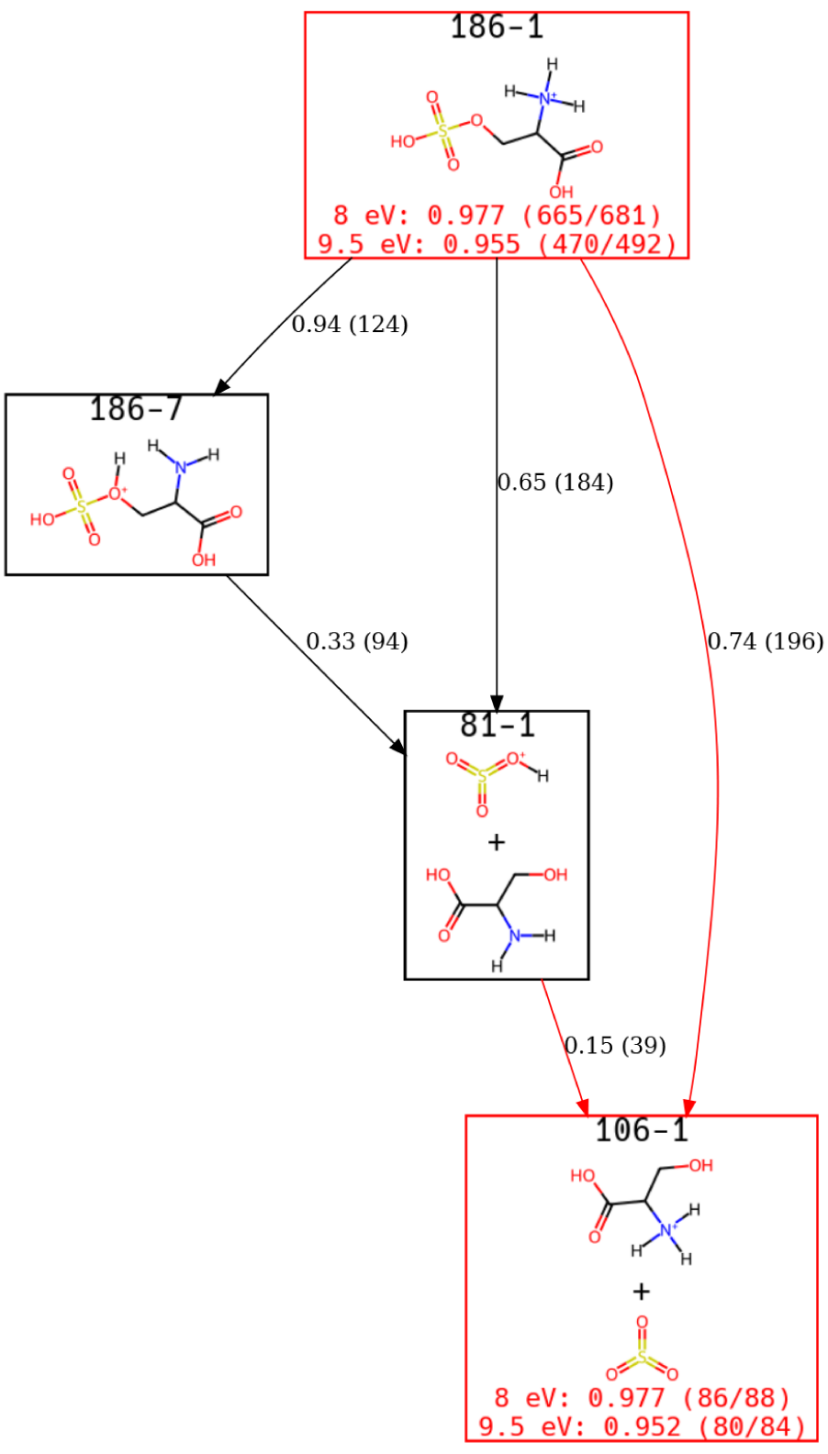
A view of the s-Ser-H^+^ ERG focusing on states identified for the *m*/*z* 106 peak. These results are consistent with previous analysis techniques but are obtained automatically using the approaches described in Section 2. Red edges represent irreversible steps, while black edges are reversible but favor the direction indicated.

Turning our attention to *m*/*z* 88, we obtain the view of the ERG shown in Figure 4. This figure highlights that a view of an ERG does not change the underlying data but rather changes how much of it is visible. For example, three pathways form the 88–3 species: a direct pathway from the starting material, one through the intermediate 106–1 state, and rarely through 88–1. Examining the vertices needed to reach 106–1, it is seen that the same information is in Figure 3, but now 106–1 is not labeled as a final product of interest but rather is itself an intermediate that accounts for roughly 25% of the 88–3 incoming flux. Looking at 186–2 and 186–7 shows that an edge is drawn, even though its contribution to the net reactive flux is small. This edge is included to show what reactions can happen among the states determined to be important. Knowing which states can interconvert can be interesting, even if they are rare events within the time scale of the simulation.

**Figure 4:**
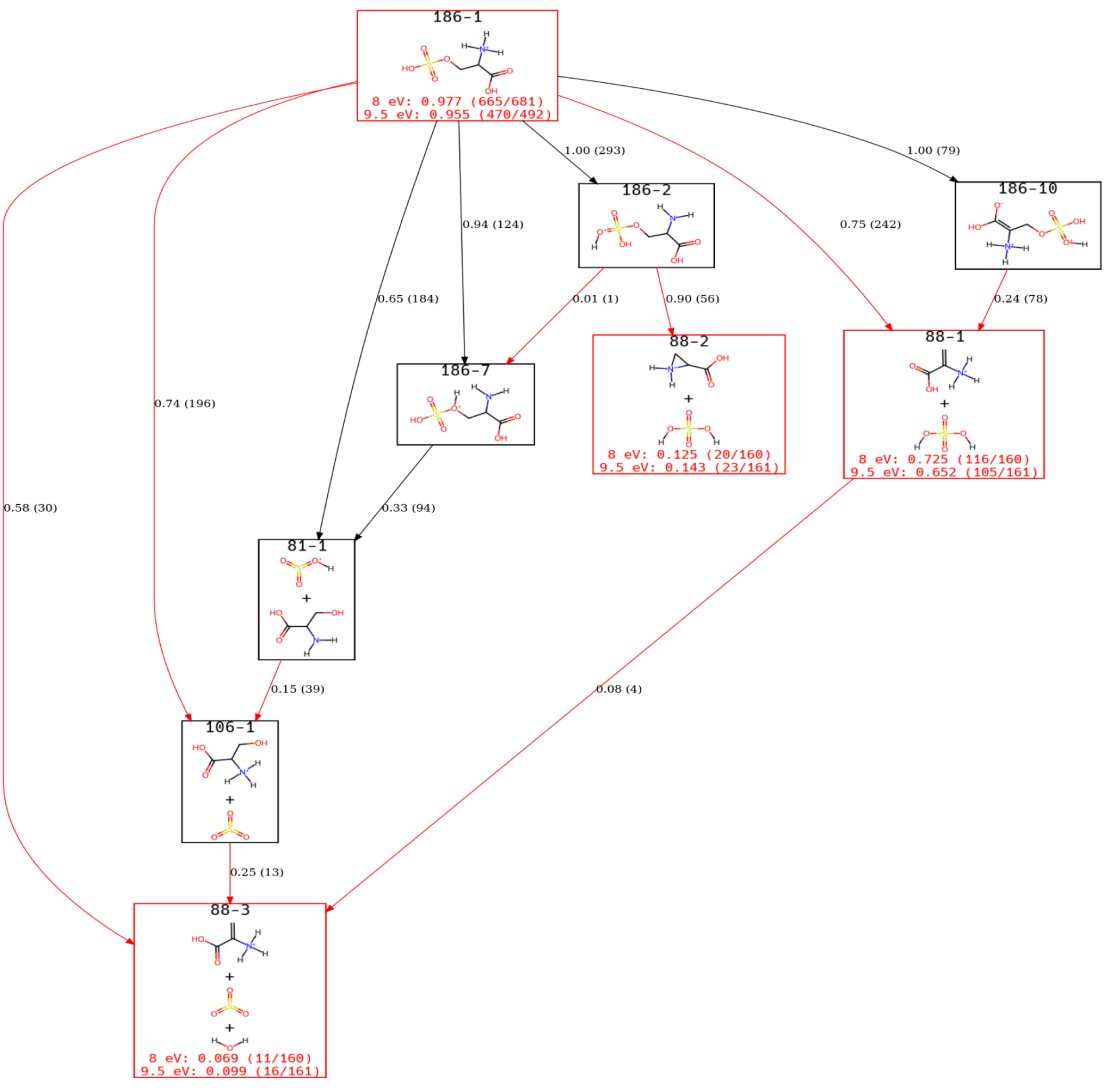
A view of the s-Ser-H^+^ ERG focusing on states identified for the *m*/*z* 88 peak. It should be noted that the node labels are automatically generated and do not fully agree with the human decisions made by Lucas et al.; in particular, 88–3 here is not 88–3 in that work.

The automatic procedures used in this work identify the three states of the system that are most important for the cutoff values selected. It should be noted that 88–1 and 88–3 share the same charged fragment, and as species were grouped by charged fragment in the previous work, these two would have been reported within the same category. The prior work found three important charged structures, while the present work found only found two. This is due to the range of collision energies considered: the third structure was only significant at a collision energy of 11 eV. That structure is still found at 8 and 9 eV and identified by the present approach, but does not appear in this view of the ERG. During follow-up DFT calculations, a fourth structure was found in the prior work, but it did not appear in the direct dynamics simulations and, hence, is not expected to be seen here. Figures 2–4 highlight the ability of the techniques present here to distill an ensemble of direct dynamics simulations into a relatively small number of mechanistic steps.

### 3.3 Lysine-H^+^ + Ar CID

To increase the chemical complexity of species studied through computational means, Lucas et al.^35^ simulated the CID of lysine-H^+^ (Lys-H^+^ - [C_6_H_15_N_2_O_2_]^+^ - *m*/*z* 147). In these simulations, the Lys-H^+^ molecule is prepared with an initially elevated microcanonical internal energy distribution, i.e., the state in which the system could be found following equilibration from one or more collisions with Ar. The most common decomposition pathways in the simulations are loss of NH_3_ to form *m*/*z* 130 ([C_6_H_12_NO_2_]^+^), loss of NH_3_ + CH_2_O_2_ to form *m*/*z* 84 ([C_5_H_10_N]^+^), and loss of CH_2_O_2_ to form *m*/*z* 101 ([C_5_H_13_N_2_]^+^). In simulations, CH_2_O_2_ was most commonly seen as C­(OH)_2_; however, that species would likely rearrange to CO + H_2_O with sufficient time. Here, we examine a subset of trajectories from the 300 kcal/mol internal energy distribution simulations. The most commonly observed peak in simulations is *m*/*z* 101, which was found to be an intermediate for *m*/*z* 84. Based on final simulation populations, four *m*/*z* 101 structures are identified. Figure 5 shows a view of the ERG explaining the reaction pathways that result in these structures.

**Figure 5:**
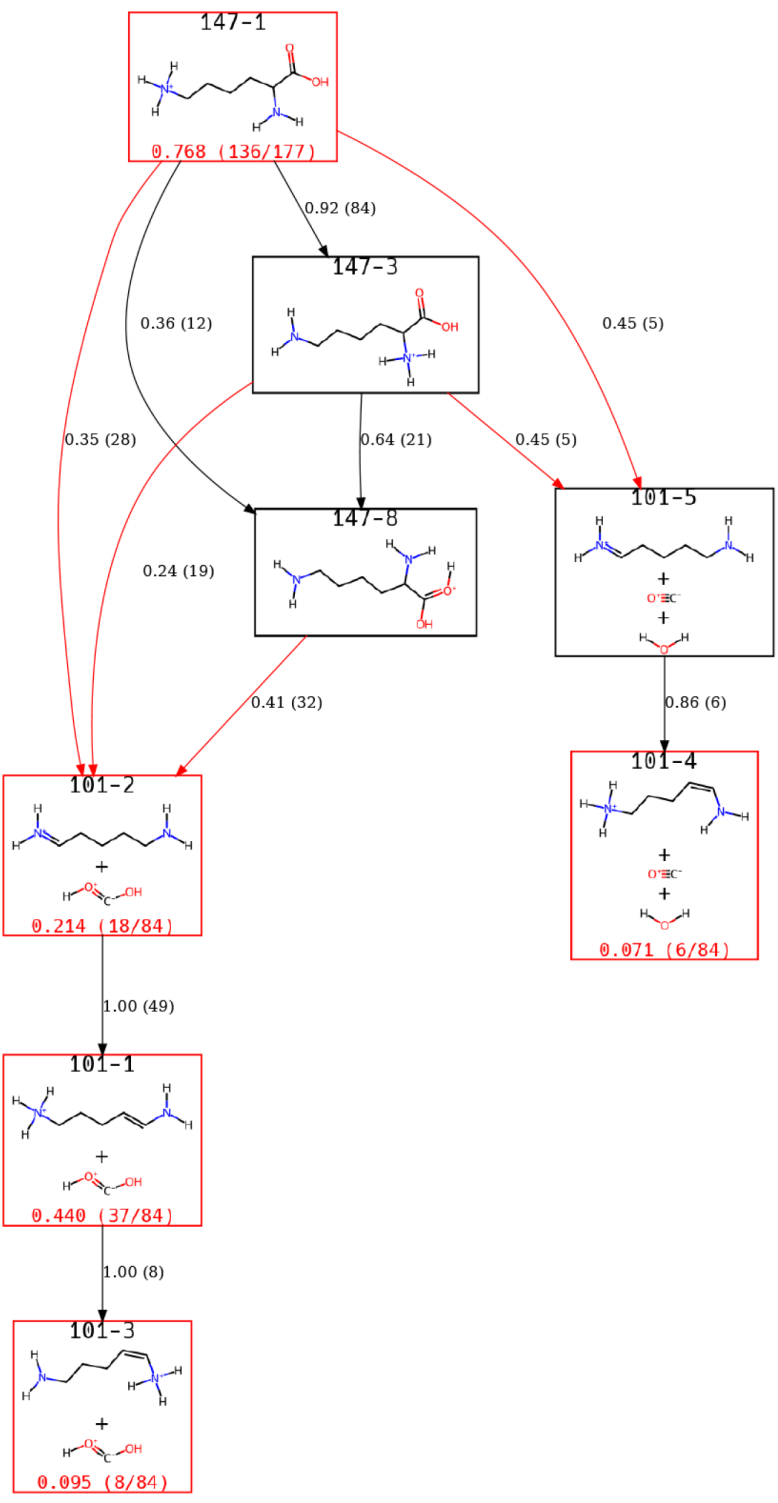
A view of the Lys-H^+^ ERG focusing on states identified for the *m*/*z* 101 peak.

This view of the ERG highlights that 101–1 through 101–3 can all interconvert with 101–2 serving as a gateway into these states. A fourth state results from the direct loss of water and CO through intermediate 101–5 and its rearrangement to 101–4. It should be noted that the charged fragments within 101–4 and 101–1 are identified as unique due to the neutral loss products and would have been considered together in the previous work. The *m*/*z* 130 was also frequently observed in simulations and found to be an intermediate to *m*/*z* 84. For *m*/*z* 130, two species are considered important at the end of the simulations with Figure 6 showing the view of the ERG for these ions.

**Figure 6:**
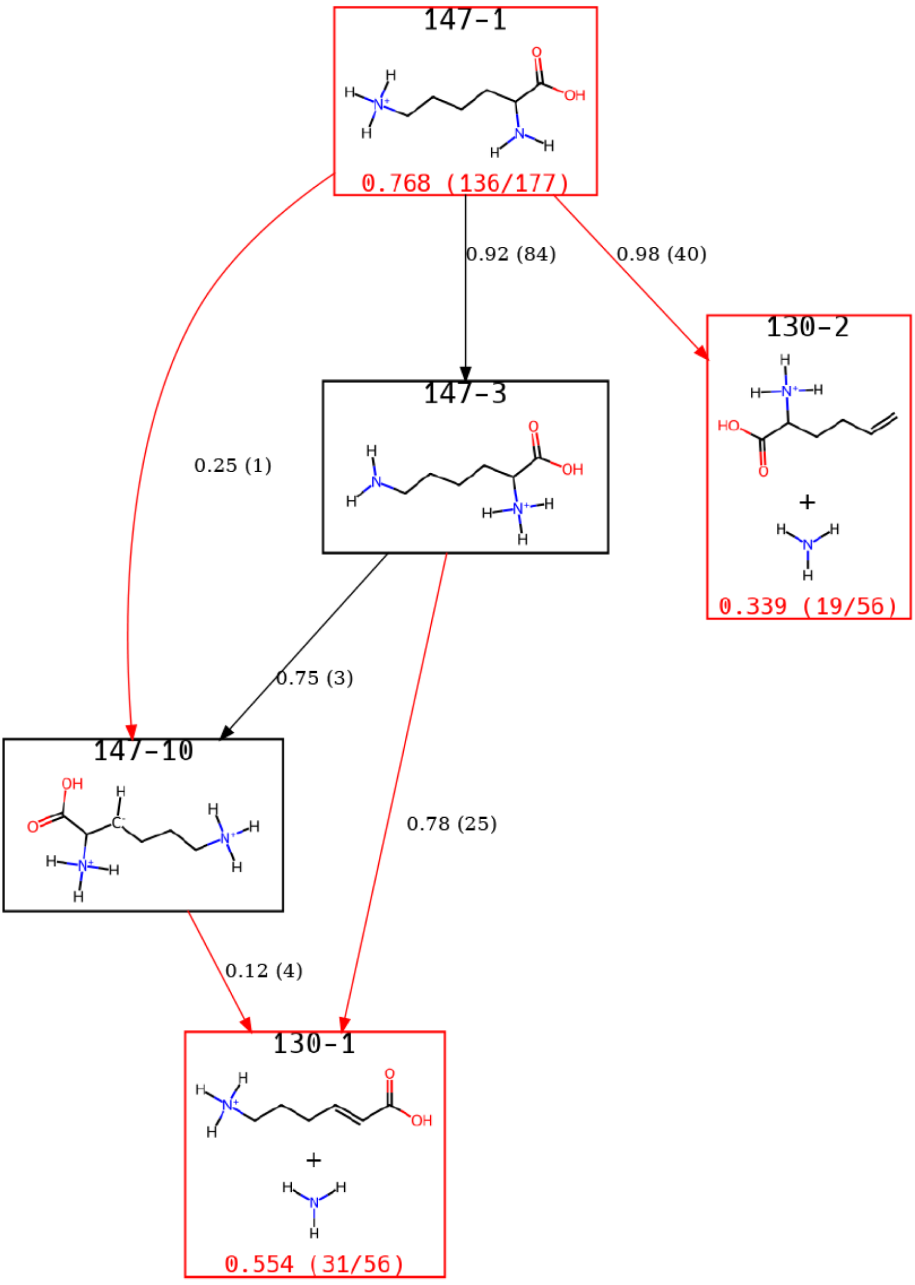
A view of the Lys-H^+^ ERG focusing on states identified for the *m*/*z* 130 peak, which results from the loss of NH_3_.

Two primary pathways are observed: the direct loss of the side-chain nitrogen results in 130–2 compared to the loss of the N-terminus, most commonly through a proton transfer from the side-chain nitrogen to the N-terminus. The two structures that arise from the loss of NH_3_ from either the side chain or the N-terminus align with the most common structures seen in simulations in prior work. We note that the percentages shown here differ and that two additional *m*/*z* 130 structures were seen in the prior work; however, we are considering a subset of that work’s data set.

Since *m*/*z* 84 is a final product that must go through two relatively stable intermediates, the population of the peak in the direct dynamics simulations was lower. As such, we determined that there were three relevant ions to consider. Two additional structures would have been included at a lower population threshold, but only six additional trajectories were observed. The view of the ERG for *m*/*z* 84 is given in Figure 7.

**Figure 7:**
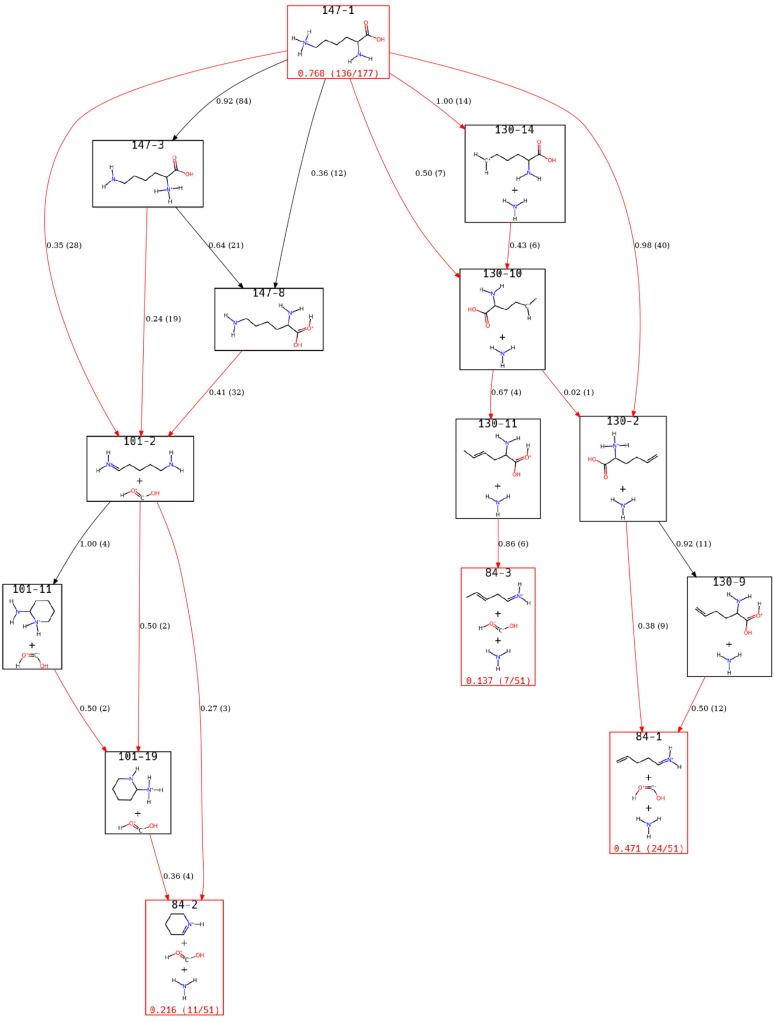
A view of the Lys-H^+^ ERG focusing on states identified for the *m*/*z* 84 peak.

Given that *m*/*z* 130 and 101 are both intermediates for *m*/*z* 84, similar states are observed in this view of the ERG and in Figures 5 and 6. The ERG shows that the formation of *m*/*z* 84 can occur either through the loss of NH_3_ followed by the loss of CO_2_H_2_ or through an initial loss of CO_2_H_2_ followed by the loss of NH_3_. In either scenario, it is likely that C­(OH)_2_ would rearrange into water and CO over sufficient time.

### 3.4 Acetyl-Lysine-H^+^ + Ar CID

Lucas et al. also studied the effect of post-translational modification on the CID of acetyl-lysine-H^+^ using a combined experimental and direct dynamics simulation approach.^37^ Experimentally, the most commonly observed decomposition pathways are *m*/*z* 126 and *m*/*z* 84. *m*/*z* 126 corresponds to the loss of NH_3_ + H_2_O + CO, while *m*/*z* 84 is formed through two different pathways: namely, through the subsequent loss of ketene from *m*/*z* 126 or from *m*/*z* 143 via the loss of acetamide. Here, we analyze a subset of simulations, specifically an ensemble with an internal energy of 300 kcal/mol and additional simulations of the primary *m*/*z* 143 decomposition product with an internal energy of 250 kcal/mol. These additional simulations were necessary since the dynamics that occurred in the initial simulations are slower than those observed in the previous two examples. By performing ″Pseudo MS3″ simulations, additional decomposition dynamics is obtained. Pseudo MS simulations are performed by starting a new ensemble of trajectories from the most commonly occurring decomposition product (143–1).

Our simulations found that acetyl-lysine-H^+^ formed *m*/*z* 143 via loss of C­(OH)_2_ (or H_2_O + CO) as shown in Figure 8. This view of the ERG considers all final products with a sizable population, regardless of *m*/*z*. Notably, the only states shown in this view of the ERG have *m*/*z* 189 or 143, i.e., a different protonation state of the initial configuration or the loss of C­(OH)_2_. Acetylation slowed the reaction dynamics sufficiently such that a single simulation could not provide information regarding the pathways observed in the experiment. Most trajectories that reacted ended in 143–1, resulting primarily from a proton transfer from the N-terminus to the C-terminus.

**Figure 8:**
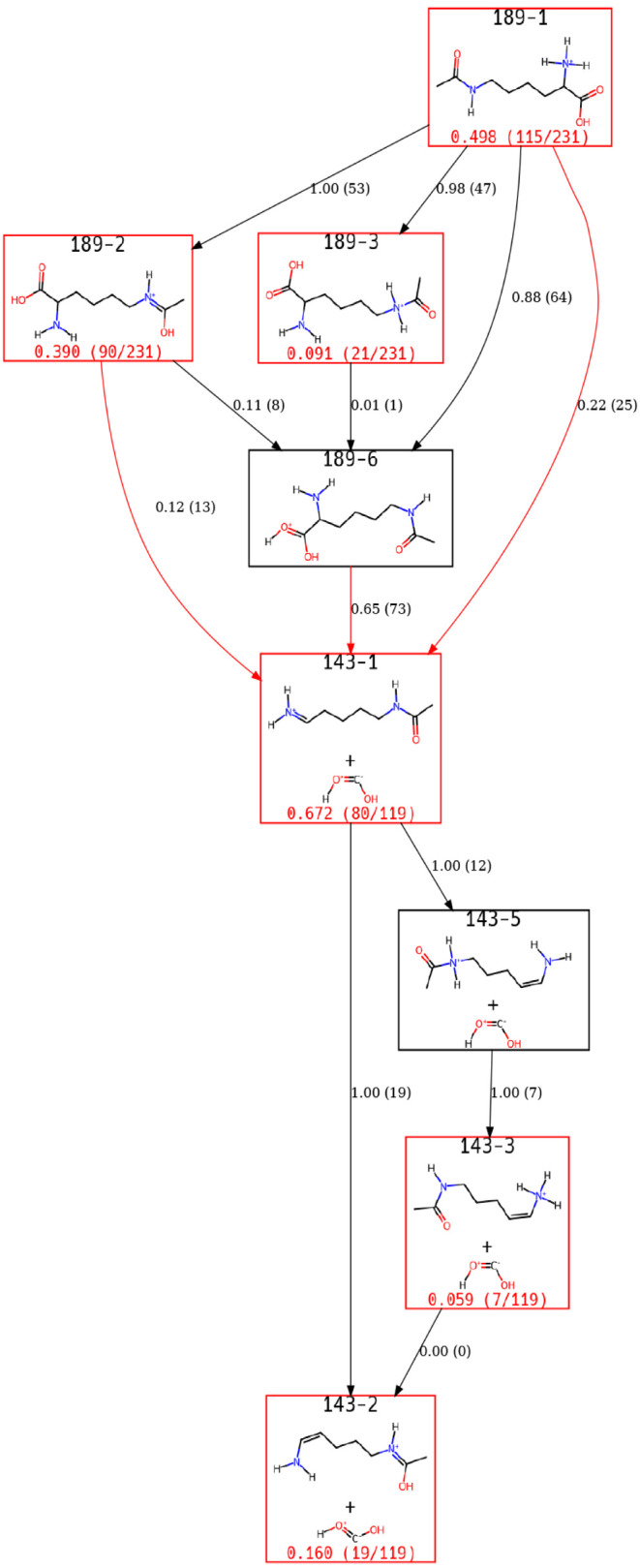
A view of the acetyl-lysine-H^+^ ERG for all ions with significant population at the end of the simulations regardless of *m*/*z*.

Turning our attention to the pseudo-MS3 simulations, the most populous peaks observed are *m*/*z* 101, 84, 43, and 126. Here, we present views of the ERG that provide insight into *m*/*z* 84, 101, and 43. The primary pathway for forming *m*/*z* 84 is via a direct loss of acetamide, as shown in Figure 9. Upon first examination of this ERG, we noted that there were some repeated Lewis structures with different ACLs. This is a result of converting nonequilibrium structures to RDKit molecules strictly using formal charge and Cartesian coordinates. It results in some ACLs being converted to the same molecule as determined by RDKits implementation of the Morgan fingerprint. For this system, these ACLs had the same *m*/*z* value, and condensing them into a single label did not change the interpretation of the ERG while also providing a more compact view. Hence, we are presenting ERGs that use these condensed labels. We note that this may not always be true and that, in the event the imperfect conversion of ACLs to RDKit molecules becomes important, some human intervention may be needed for certain systems.

**Figure 9:**
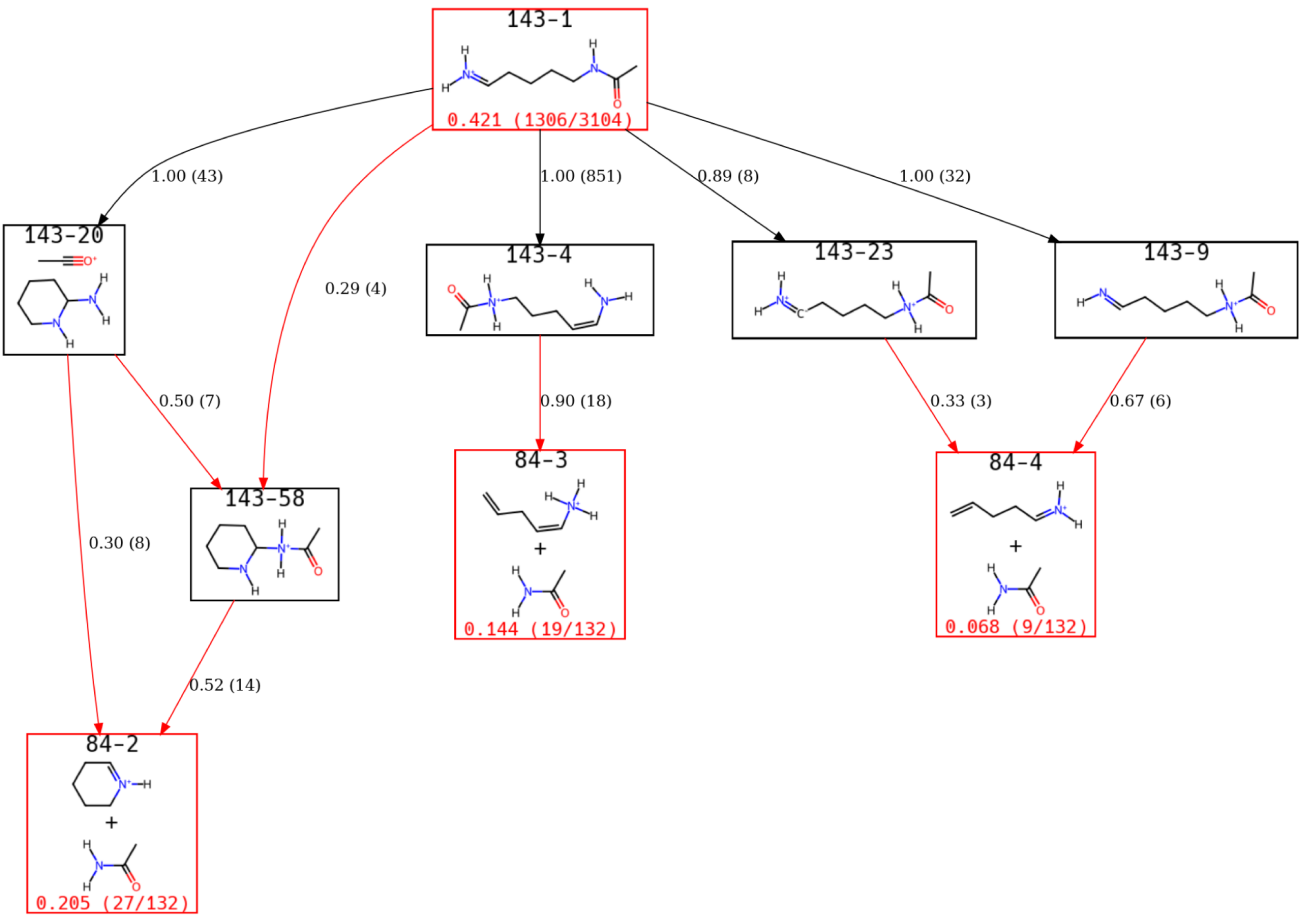
A view of the ERG starting from *m*/*z* 143 and focusing on the direct loss of acetamide to produce *m*/*z* 84.

*m*/*z* 84 can also form via an indirect process, as shown in Figure 10. In our prior work, we identified that this primarily occurs through an *m*/*z* 126 intermediate, which is true; however, the view of the ERG presented in Figure 10 illustrates that this pathway is more nuanced. Based on current thresholds, a pathway involving *m*/*z* 101 is also important, highlighting the value added through this method of analysis.

**Figure 10:**
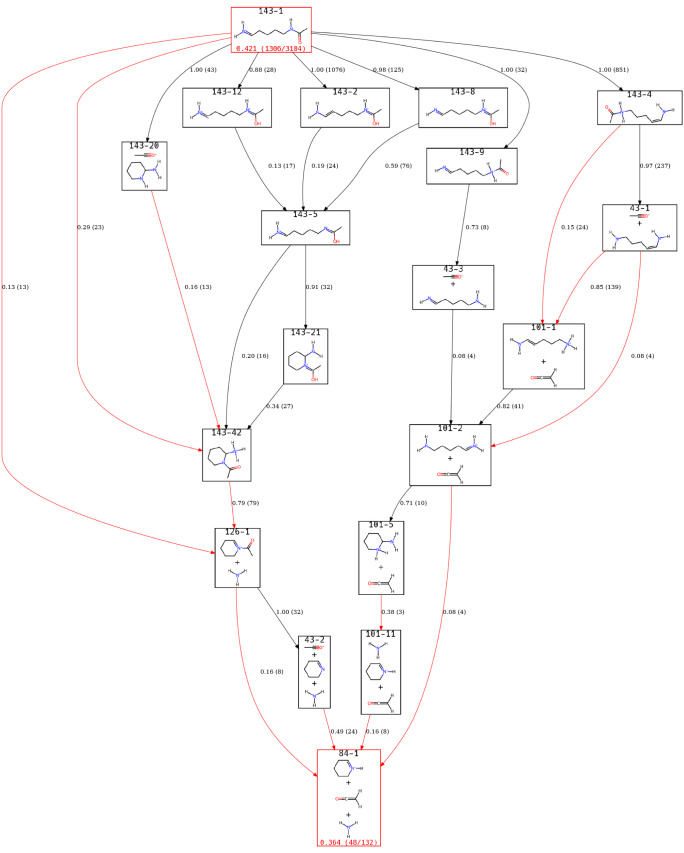
A view of the ERG starting from *m*/*z* 143 focusing on indirect mechanisms of reaching *m*/*z* 84.

Figure 11 shows a view of the ERG that focuses on the commonly observed peaks at *m*/*z* 101 and 43. From the above, the relevance of *m*/*z* 101 to the experimentally observed peak of *m*/*z* 84 is clear. This figure also illustrates the interrelation of *m*/*z* 101 and *m*/*z* 43, i.e., that *m*/*z* 101 is the loss of ketene, while *m*/*z* 43 is the formation of protonated ketene. The most commonly occurring state is 101–1, which is primarily formed via 43–1, an intermediate step forming protonated ketene, followed by a proton transfer back to the larger fragment. This analysis method identifies steps that may occur via noncovalent complexes or roaming mechanisms.

**Figure 11:**
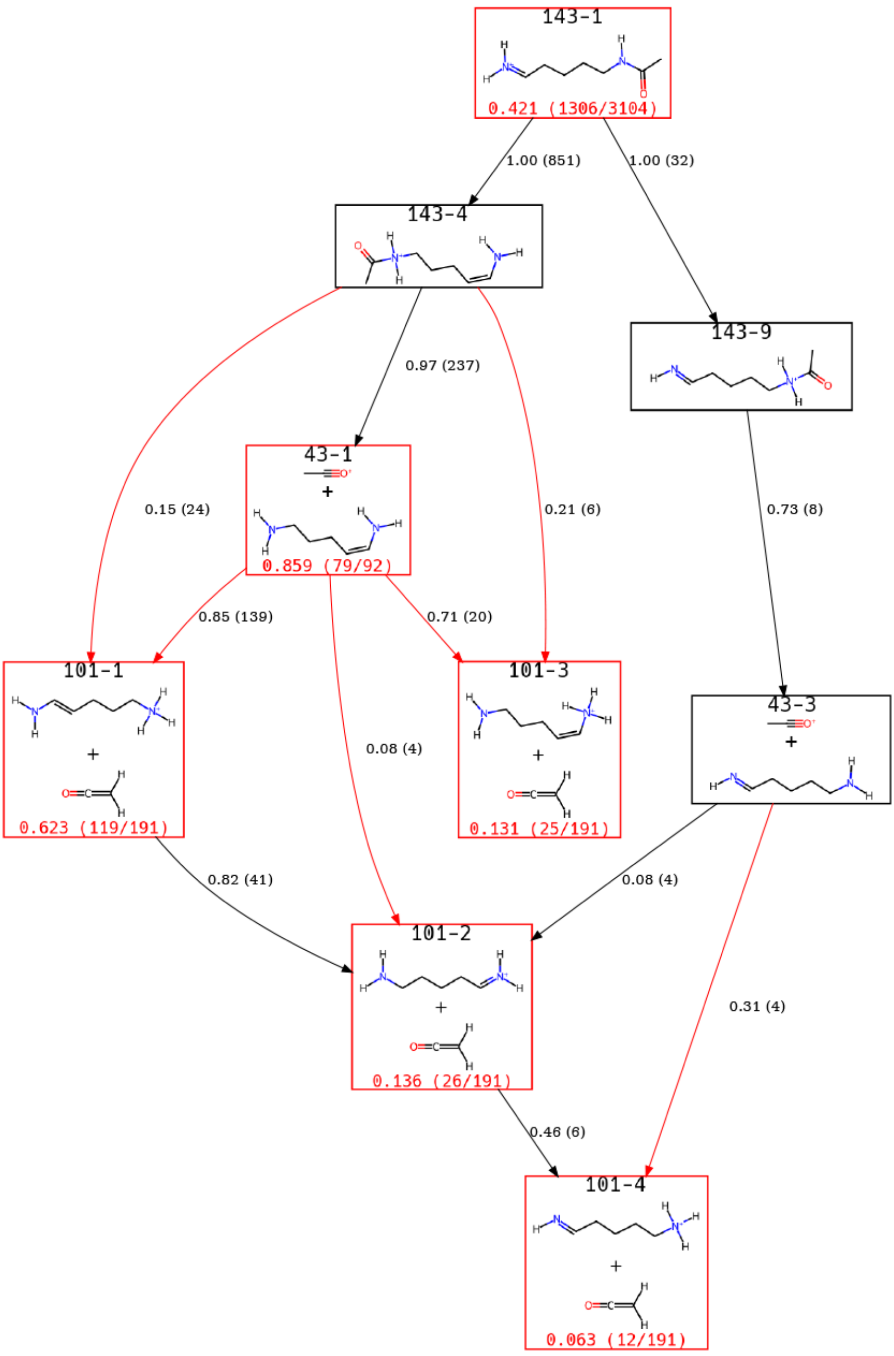
A view of the ERG for the pseudo MS3 simulations focused on *m*/*z* 101 and 43.

## 4 Conclusion

Graph theory provides powerful analysis tools that can streamline the analysis of direct dynamics simulations. Here, we have proposed an extension to the analysis framework established by Perez Mellor and Spezia to allow for more consistent labeling of nonequilibrium structures. In particular, we proposed a means to include collective chemical properties into the graph at the node level. These collective chemical properties are represented by including one or more abstract nodes that provide information regarding which group of atoms currently holds the collective chemical property. By adding these additional nodes, it both provides the graph with more information and preserves the information already contained within the graph related to the atomic connectivity. These augmented molecular structure graphs allow for the determination of augmented canonical labels that encode not just the connectivity but also the (dis)­connected component that holds the collective property. This, in turn, allows for a more straightforward identification of the relevant chemical states visited during a direct dynamics simulation.

By performing a time series analysis that makes use of isomorphism tests between frames, one can identify the relevant chemical states that are visited throughout a trajectory, which provides a compact summary of the reactivity that occurred for a single trajectory. Obtaining this information for an ensemble of trajectories allows us to form an ensemble reaction graph (ERG), which contains information about all states visited and which states can interconvert. Since this ERG contains a summary of the chemically relevant transitions for the entire ensemble, it quickly becomes difficult to visualize. To address this challenge, we describe an approach to obtain filtered views of the ERG that provide insight into the most commonly occurring pathways that lead to a set of chemical states of interest. The addition of RDKit allows for an automatic means of viewing the most probable reaction mechanisms for the entire ensemble. The augmented molecular structure graph and filtered ensemble reaction graph framework allow for a more efficient evaluation of results from direct dynamics simulations for diverse systems. This was illustrated for three previously reported systems. Not only were the previous results reproduced automatically, but this new method also provides additional information since the augmented canonical labels offer information about both the ion and neutral species simultaneously.
